# Dr. George Papanicolaou: The Visionary Who Revolutionized Women’s Health

**DOI:** 10.7759/cureus.69302

**Published:** 2024-09-12

**Authors:** Maria Nikoloudi, Kyriaki Mystakidou

**Affiliations:** 1 Pain Relief and Palliative Care Unit, Department of Radiology, Areteion Hospital, School of Medicine, National and Kapodistrian University of Athens, Athens, GRC

**Keywords:** cervical cancer, cytology, historical vignette, pap smear, women's health

## Abstract

Dr. George Papanicolaou, a distinguished Greek physician, biologist, and researcher, made monumental contributions to medical science, particularly in the field of cytopathology. His groundbreaking work in the early detection of cervical cancer through the development of the Pap smear has had an enduring global impact, transforming women's healthcare and significantly reducing mortality rates associated with cervical cancer. Papanicolaou's journey from his early education in Greece to his pioneering research in the United States exemplifies a relentless pursuit of scientific discovery and innovation. Papanicolaou's life and achievements continue to serve as a beacon of innovation in medical research, illustrating the profound impact that one individual’s dedication can have on public health and the ongoing fight against cancer.

## Introduction and background

Dr. George Papanicolaou stands as one of the most influential figures in modern medical history, particularly for his pioneering work in the early detection of cervical cancer. Born in a small town in Greece, Papanicolaou's journey from a young student with a passion for medicine to a globally recognized scientist is a testament to his relentless dedication and visionary approach. His name is synonymous with the Pap smear, a simple yet revolutionary test that has saved millions of lives by enabling the early detection and treatment of cervical cancer [1].

However, Papanicolaou's contributions to medical science extend far beyond the development of this life-saving test. His innovative research in cytopathology laid the foundation for modern cancer screening methods and transformed the way the medical community approaches the diagnosis and prevention of various cancers [2]. Papanicolaou's work not only had a profound impact on women’s health but also catalyzed significant advancements in public health policies and preventive care practices worldwide.

In a time when cancer was often a death sentence, Papanicolaou's work provided a beacon of hope. He demonstrated that early detection could dramatically improve patient outcomes, a concept that has since become a cornerstone of modern medicine [3]. His story is not just one of scientific achievement but also of perseverance, as he overcame skepticism and numerous challenges to bring his vision to fruition.

This review aims to explore the life and legacy of Dr. George Papanicolaou, delving into the scientific journey that led to his groundbreaking discoveries, the impact of his work on global health, and the challenges and controversies that have surrounded the Pap smear over the decades. By examining the broader context of his contributions, we can better appreciate the enduring significance of his work in shaping the landscape of modern healthcare.

Early life and education

George Nicholas Papanicolaou, one of the most significant figures in the history of medical science, was born on May 13, 1883, in the small town of Kymi on the island of Euboea, Greece. His early life was deeply influenced by his family, particularly his father, Nicholas Papanicolaou, a well-respected physician in their community. Growing up in an environment where the importance of medical practice and patient care was a daily conversation, George was naturally drawn to the field of medicine from a very young age. This early exposure to the medical profession not only sparked his interest in human biology but also instilled in him a profound sense of duty to contribute to the well-being of others.

**Figure 1:**
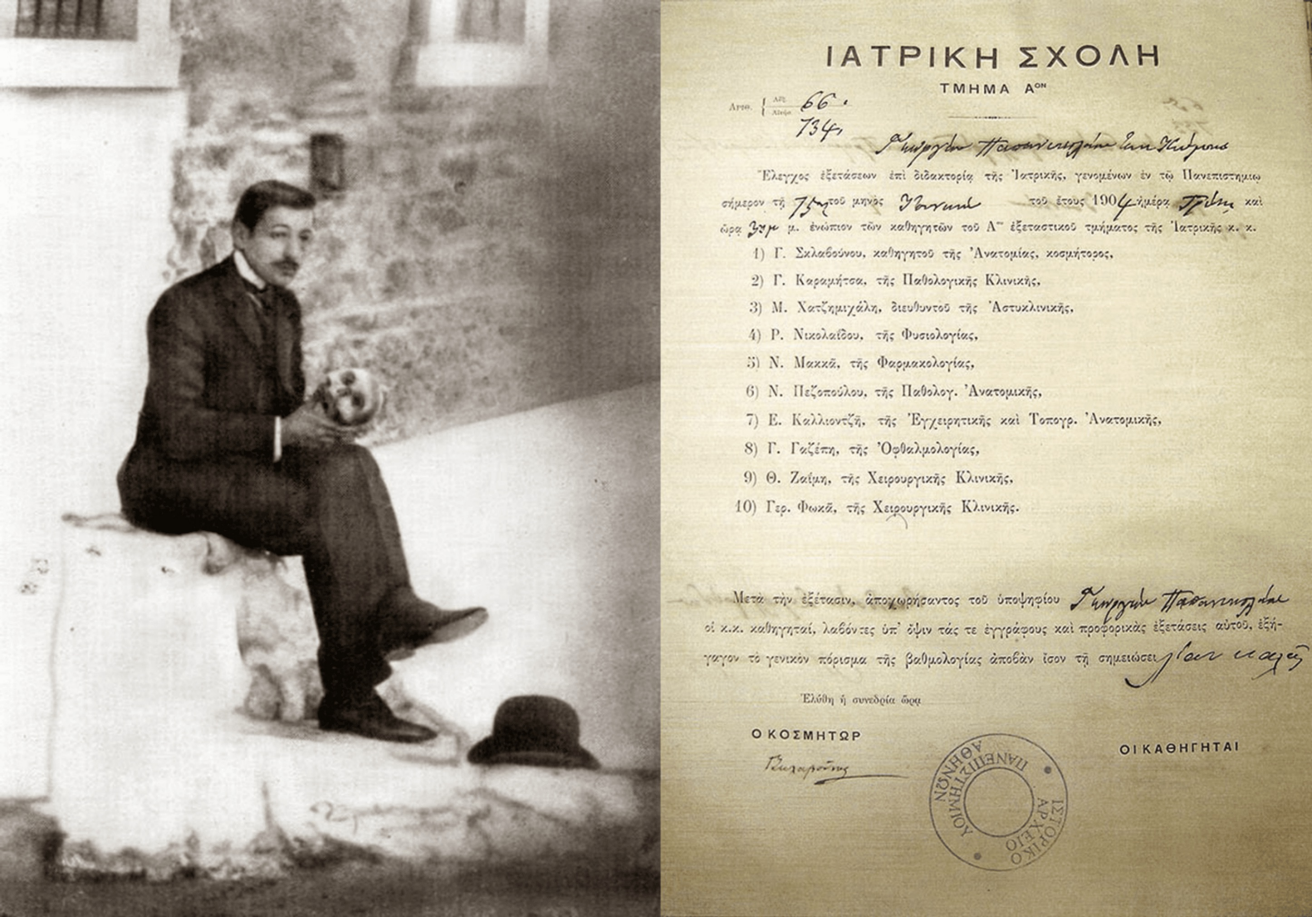
The young George Papanicolaou and his degree from the medical school of Athens Figure permissions were obtained from the Hellenic Literary and Historical Archive (ELIA) and Ms. Giouli P. Kokkori.

As a student, Papanicolaou demonstrated exceptional academic abilities. His intellectual curiosity and dedication to his studies were evident during his years at the University of Athens, where he pursued a medical degree. He graduated in 1904, at the age of 21, a remarkable achievement that underscored his commitment to becoming a physician [4]. During his time at the University of Athens, Papanicolaou was known for his inquisitive nature and his desire to understand the complexities of human anatomy and pathology. His education at this prestigious institution provided him with a robust foundation in medical sciences, which would later prove invaluable in his pioneering research (Figure 1).

Further studies and early career

Papanicolaou's thirst for knowledge did not end with his medical degree. After completing his studies in Athens, he sought to broaden his horizons by pursuing further education abroad, a decision that would greatly influence the trajectory of his career. He moved to Germany, a hub of scientific research and innovation at the time, to continue his studies. In Germany, Papanicolaou attended the University of Munich, where he studied under some of the most prominent scientists of the era. He immersed himself in advanced research, focusing on biology and pathology, and in 1910, he earned a PhD from the University of Munich. This period in Germany was crucial in shaping his scientific approach and methodology, as he was exposed to cutting-edge research techniques that would later inform his work in cytopathology [5].

Papanicolaou's experiences in Germany were not limited to academic achievements; they also broadened his cultural and intellectual perspectives. Living in a country at the forefront of scientific discovery, he was inspired by the rigorous research environment and the collaborative spirit among scientists. These experiences reinforced his belief in the power of research to drive medical advancements and fueled his determination to contribute to the field [5].

After completing his doctorate, Papanicolaou returned to Greece with the intention of applying his knowledge to improve medical practices in his homeland. However, his return coincided with the Balkan Wars of 1912-13, and like many of his contemporaries, he was drawn into the conflict. Papanicolaou served as a physician in the Greek military, where he gained first-hand experience in the challenges of medical care in wartime. This period of service not only tested his medical skills under pressure but also deepened his understanding of human suffering and the urgent need for medical innovations that could save lives [6].

Following the war, Papanicolaou realized that his aspirations for a career in medical research would be better fulfilled abroad, given the limited opportunities in Greece at the time. In a letter to his father, he wrote: "No, I don't want to be a military doctor. I want to be free, to feel the joy that the struggle of life gives. I'm not afraid of the ocean. I want my freedom, my sweet freedom" [6].

**Figure 2:**
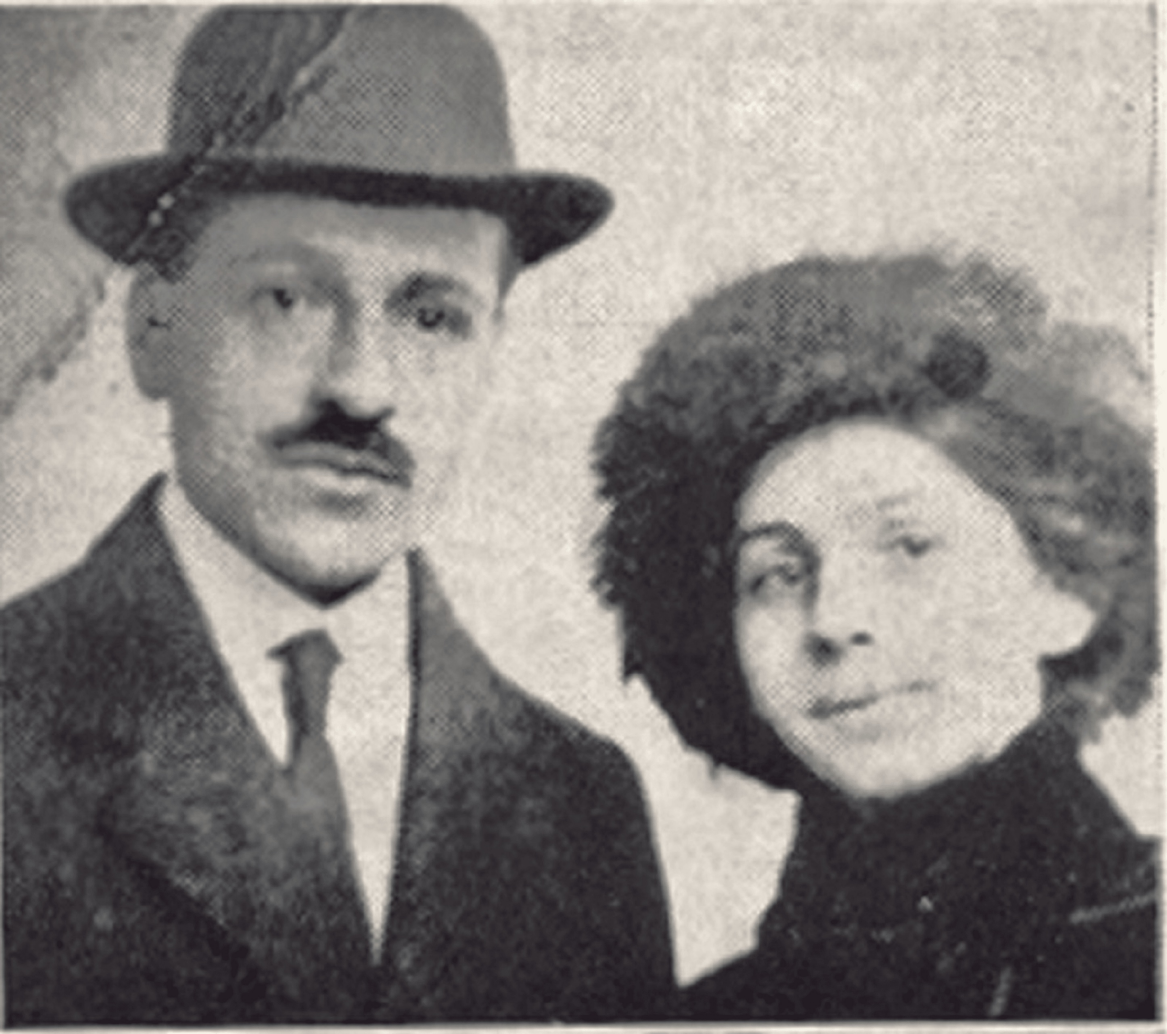
George Papanicolaou and his wife Andromachi (Mary) Mavrogeni Figure permissions were obtained from the Hellenic Literary and Historical Archive (ELIA) and Ms. Giouli P. Kokkori.

In 1913, shortly after his marriage to Andromachi (Mary) Mavrogeni, Papanicolaou and his wife made the life-changing decision to emigrate to the United States. This move marked the beginning of a new chapter in his life, one that would eventually lead to his most significant scientific contributions [6] (Figure 2).

Upon arriving in the United States, Papanicolaou initially faced numerous challenges, including financial difficulties and the need to establish himself in a new country. However, his determination and passion for research soon led to an opportunity at Cornell University Medical College in New York City. In 1914, he was appointed as a researcher in the Department of Anatomy at Cornell, a position that allowed him to pursue his scientific interests while working alongside some of the leading medical professionals of the time [4].

At Cornell, Papanicolaou's early research focused on the reproductive physiology of guinea pigs, particularly the estrous cycle, which laid the groundwork for his later discoveries in cytopathology [7]. His meticulous approach to research and his ability to draw connections between basic biological processes and clinical applications set him apart from his peers. The skills and insights he developed during these early years at Cornell would eventually lead to his groundbreaking work on the Pap smear, a test that would revolutionize the field of cancer detection and save countless lives worldwide [8].

Thus, Papanicolaou's early life and education were characterized by a relentless pursuit of knowledge, a deep commitment to medical science, and a willingness to overcome obstacles in order to achieve his goals. His journey from a small town in Greece to the research laboratories of Cornell University reflects his enduring dedication to the advancement of medical science and his unwavering belief in the potential of research to transform healthcare.

## Review

Development of the Pap smear

**Figure 3:**
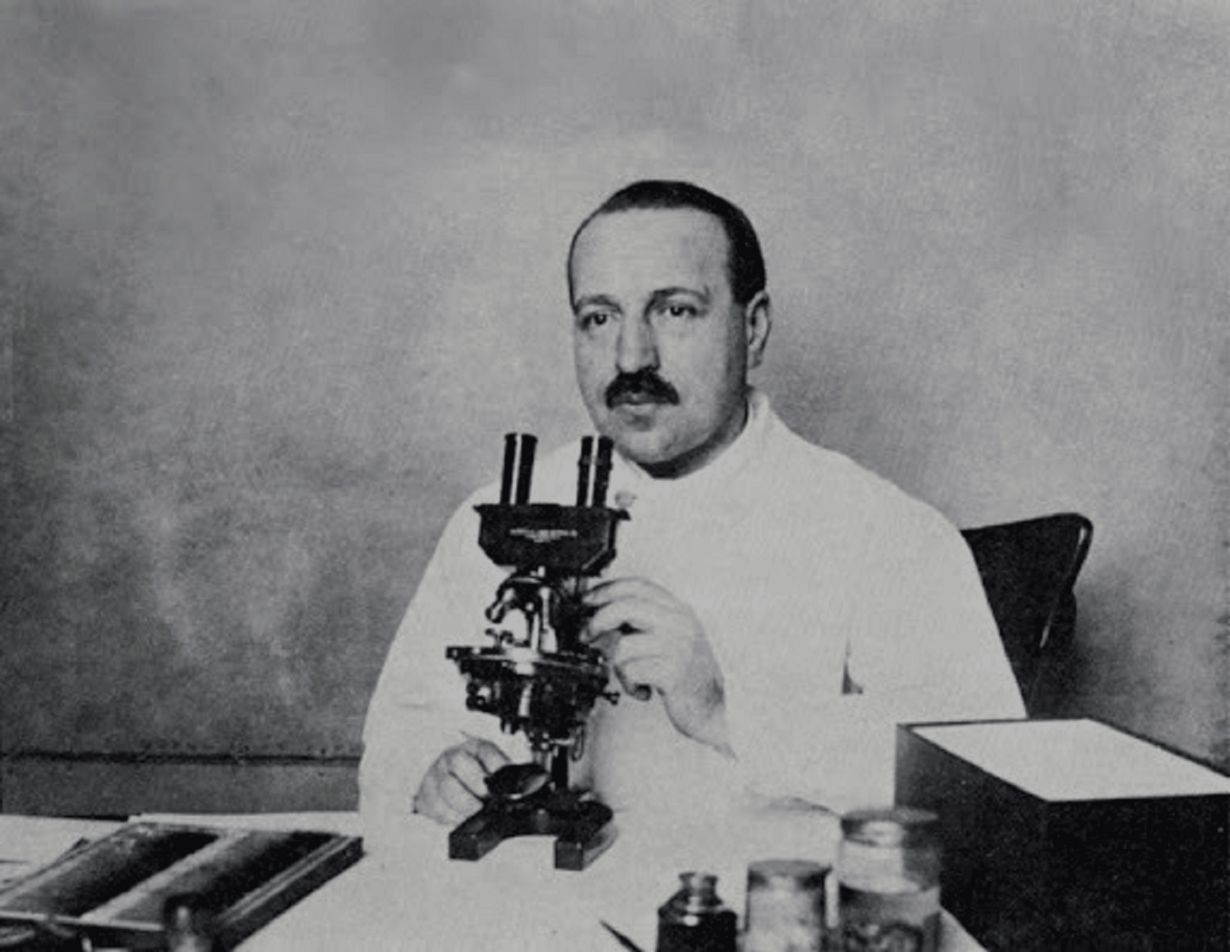
George Papanicolaou at Cornell University Figure permissions were obtained from the Hellenic Literary and Historical Archive (ELIA) and Ms. Giouli P. Kokkori.

Papanicolaou's work at Cornell University marked the beginning of his groundbreaking research in cytopathology. Initially, he conducted research on the estrous cycle of guinea pigs, which led him to observe cellular changes in the vaginal smears of these animals. These observations sparked his interest in applying similar techniques to humans to detect cellular abnormalities [5] (Figure 3).

In 1928, Papanicolaou presented his preliminary findings at a medical conference, suggesting that vaginal smears could be used to detect uterine cancer. However, his ideas were met with skepticism, and it took years of persistent research and advocacy before the medical community began to recognize the significance of his work [2].

Collaborating with Dr. Herbert Traut, a gynecologist, Papanicolaou conducted extensive studies and published their findings in 1943 in the landmark monograph "Diagnosis of Uterine Cancer by the Vaginal Smear". This publication provided comprehensive evidence of the efficacy of the Pap smear in detecting cervical cancer at an early, treatable stage [9].

Impact on women's health

**Figure 4:**
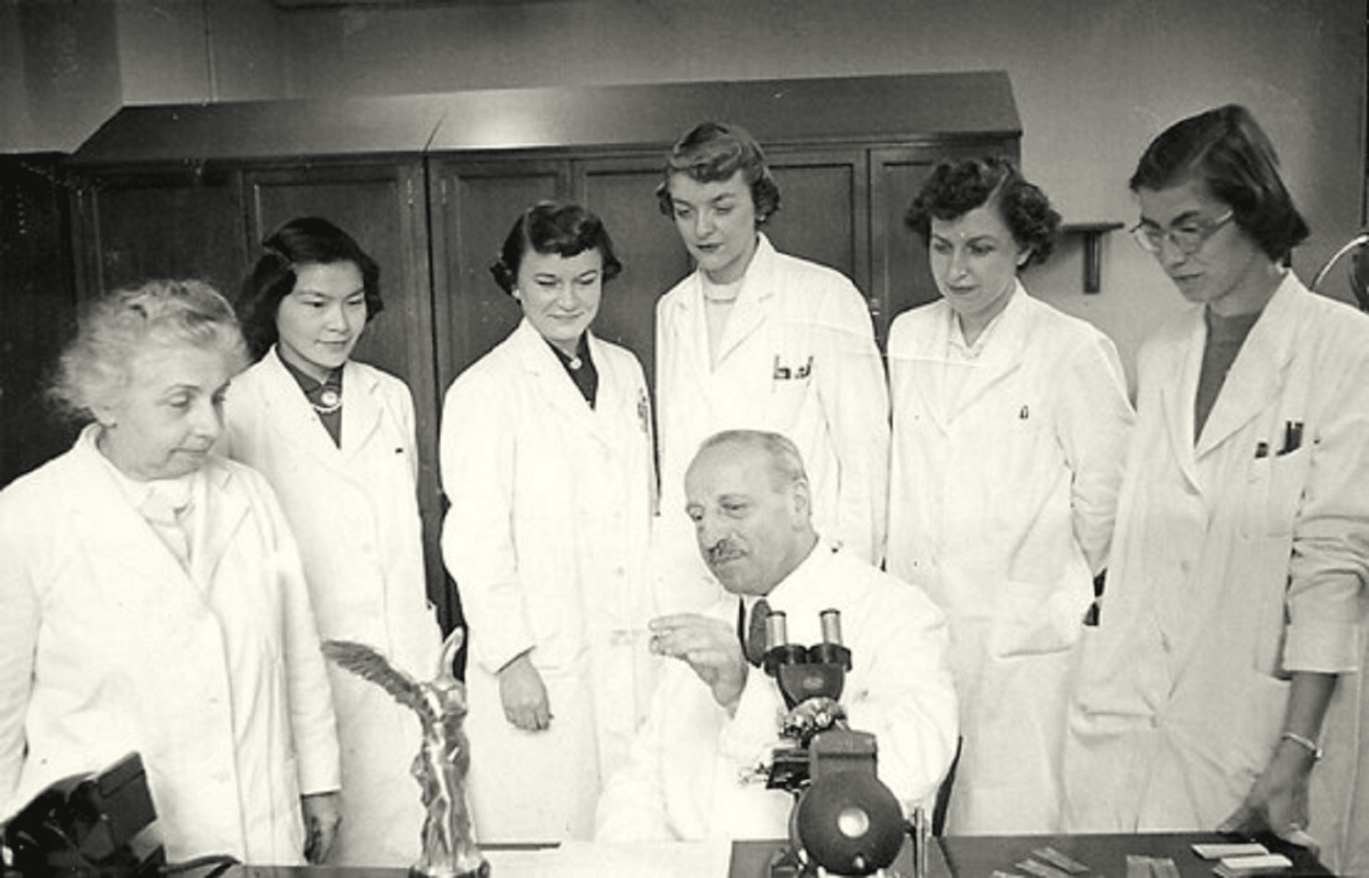
George Papanicolaou and his colleagues Figure permissions were obtained from the Hellenic Literary and Historical Archive (ELIA) and Ms. Giouli P. Kokkori.

The introduction of the Pap smear revolutionized women's health care. Before its widespread use, cervical cancer was a leading cause of death among women. The Pap smear enabled the early detection of precancerous and cancerous cells in the cervix, significantly reducing mortality rates. Public health campaigns and screening programs based on Pap smears have saved countless lives by identifying cervical cancer in its early stages when it is most treatable [3] (Figure 4).

The test's impact extends beyond cervical cancer, as it has also been instrumental in detecting other gynecological conditions. Papanicolaou's work emphasized the importance of regular screenings and preventive care, reshaping how the medical community approaches women's health [2].

Scientific and medical contributions

Papanicolaou's contributions to cytopathology and cancer detection have been widely recognized. The development of the Pap smear laid the groundwork for advances in the field of cytology, a branch of pathology that studies disease at the cellular level. His work demonstrated the potential of cytological techniques in early cancer detection, inspiring further research and innovation [10].

The Pap smear's success also highlighted the importance of interdisciplinary collaboration in medical research. Papanicolaou's partnership with Dr. Traut exemplifies how combining expertise from different medical fields can lead to groundbreaking discoveries [5].

Public health and policy implications

The widespread adoption of the Pap smear had significant public health implications. It led to the establishment of national screening programs in many countries, which have become a standard part of women's healthcare. These programs have contributed to a substantial decline in cervical cancer incidence and mortality [11].

Moreover, Papanicolaou's work has influenced public health policies related to cancer prevention and screening. Governments and health organizations worldwide have recognized the importance of early detection and have invested in education and infrastructure to support regular screenings. The success of the Pap smear has also paved the way for the development and implementation of other cancer screening tests [3].

Challenges and controversies

Despite its success, the Pap smear has faced challenges and controversies. One of the primary challenges has been ensuring access to regular screenings for all women, particularly those in underserved and low-income communities. Barriers such as lack of healthcare access, cultural stigmas, and limited awareness have hindered the reach of screening programs [12].

Additionally, the accuracy of the Pap smear has been a subject of debate. While it is highly effective, false-positive and false-negative results can occur. Ongoing advancements in cytological techniques and the introduction of complementary screening methods, such as HPV testing, aim to address these limitations and improve diagnostic accuracy [13].

## Conclusions

Dr. George Papanicolaou's pioneering work in cytopathology and the development of the Pap smear have had an enduring impact on medical science and women's health. His dedication to research and his innovative approach to cancer detection have saved countless lives and revolutionized the field of preventive care. The Pap smear remains a cornerstone of gynecological screening programs worldwide, underscoring the importance of early detection and regular screenings in reducing cancer mortality rates. His work has inspired ongoing research and advancements in cytology and cancer screening, emphasizing the critical role of interdisciplinary collaboration in medical research. The public health policies and screening programs that emerged from his discoveries continue to shape the landscape of women's healthcare, highlighting the importance of accessibility and education in preventive medicine.

As we reflect on Dr. Papanicolaou's contributions, it is clear that his work has left an indelible mark on the medical community and has profoundly improved the quality of life for women around the world. His story is a testament to the power of perseverance, innovation, and the relentless pursuit of knowledge in the service of humanity.
